# Development of a CRISPR/Cas9 system against ruminant animal brucellosis

**DOI:** 10.1186/s12917-019-2179-z

**Published:** 2019-11-27

**Authors:** Garyfalia Karponi, Spyridon K. Kritas, Gina Papadopoulou, Elissavet-Kalliopi Akrioti, Eleni Papanikolaou, Evanthia Petridou

**Affiliations:** 1 Department of Microbiology and Infectious Diseases, School of Veterinary Medicine, Faculty of Health Sciences, Aristotle University of Thessaloniki, 54124 Thessaloniki, Greece; 2 Cellular Immunology Laboratory, Basic Research, Biomedical Research Foundation of the Academy of Athens, 11527 Athens, Greece; 3 Laboratory of Cellular and Molecular Neurobiology-Stem Cells, Hellenic Pasteur Institute, 11521 Athens, Greece; 4 Laboratory of Biology, School of Medicine, National and Kapodistrian University of Athens, 11527 Athens, Greece; 5 Department of Research and Development, Miltenyi Biotec, 51429 Bergisch Gladbach, Germany

**Keywords:** Brucellosis, Gene therapy, Viral vectors, Ruminant animals, Macrophages

## Abstract

**Background:**

Brucellosis, caused by several *Brucella* species, such as the bacterium *Brucella melitensis*, is considered one of the most severe zoonotic diseases worldwide. Not only does it affect ruminant animal populations, leading to a substantial financial burden for stockbreeders, but also poses severe public health issues. For almost four decades in southern Europe and elsewhere, eradication of the disease has been based on ambiguously effective programs, rendering massive sanitation of livestock urgent and indispensable. Gene therapy, which has been proved effective in the clinic, could possibly constitute an alternative option towards a permanent cure for brucellosis, by aiding in the deletion or inactivation of genes associated with the replication of *Brucella* within the host cells.

**Results:**

We infected ovine macrophages with *B.melitensis*, to simulate the host cell/microorganism interaction in vitro, and transduced the infected cells with CRISPR/Cas9 lentiviral vectors that target *Brucella*’s RNA polymerase subunit A (RpolA) or virulence-associated gene *virB10* at a multiplicity of infection of 60. We demonstrate a significant decrease in the bacterial load per cell when infected cells are transduced with the RpolA vector and that the number of internalized brucellae per cell remains unaffected when macrophages are transduced with a conventional lentiviral vector expressing the green fluorescence protein, thus underlining the bactericidal effect of our CRISPR/Cas9 system.

**Conclusions:**

Pending in vivo verification of our findings, overall, these results may prove critical not only for the treatment of human brucellosis, but for other infectious diseases in general.

## Background

Brucellosis is an infectious, globally distributed disease, caused by bacteria of the genus *Brucella* [1]. Owing to their ability to maintain their virulence for several months, brucellae are considered to be among the most resistant, non-spore forming, Gram-negative bacteria [2]. Although the most common species, *B.melitensis*, mainly affects ruminant animals, provoking abortions and infertility [3], it may be transmitted to humans following direct contact with an infected animal or via ingestion of contaminated products [4]. This results in severe effects, such as arthritis, endocarditis, meningitis, spondylitis, epididymitis and orchitis, accompanied with sterility in men and abortion in women [1]. The disease cannot be prevented, since no effective vaccine currently exists for human use. In infected individuals, antibiotic therapy is long-lasting and treatment may lead to relapse or re-infection after de novo exposure to the microorganism [1].

It is well-established that once infected, sick animals shed large numbers of *Brucella* in semen, fetal fluids and vaginal exudates [3]. Brucellosis’s pathogenicity resides in the microorganism’s ability to maintain its virulence for many months, even outside a host’s body and under extreme environmental conditions [3], thus critically contributing to the spread of the disease. In non-immunized animals, brucellae may escape the phagocytic activity of macrophages and proliferate inside them [5], thus infecting not only the tissues of the reticuloendothelial system, but also reproductive organs [6].

Currently, vaccination programs in livestock, frequent monitoring and slaughtering of seropositive carriers constitute the only available means for eradicating the disease [7]. Although cellular immunity mechanisms are employed in immune animals to destroy brucellae [1], vaccination and supervision of herds are often ineffective. The REV-1 vaccine, despite its vast use for the prevention of brucellosis in sheep and goats [8], may cause abortions and infertility in pregnant and male animals respectively [9], leading to its extensive diffusion into the environment with vaginal discharges during post-partum period. In addition, since it partially preserves its virulent properties, it is not entirely safe for clinical practitioners who administer the vaccine [9]. Furthermore, the wildtype strain in seropositive animals is not serologically distinctive from the vaccine strain. This, in combination with the extensive immune reaction provoked by REV-1 occasionally, pose a major hurdle in discriminating the vaccinated from the truly infected animals [10]. In cattle, even though vaccination with REV-1 is applied in certain cases [11], its suitability has not been clarified by the manufacturers for these animals.

Although brucellosis is no longer a threat in northern European countries and USA/Canada, it has not been yet eradicated in countries along the Mediterranean, parts of Africa and Asia, the Middle East and Central and South America [2]. It is worth-noting that application of programs for the eradication of brucellosis have been failing in southern Europe for almost four decades [12]. Consequently, *Brucella* infection in livestock, not only poses severe public health issues, but also translates into a substantial financial burden for stockbreeders, mainly due to the abortions [13].

The urgent need for massive sanitation of livestock may be covered by gene therapy, a much promising strategy employing incorporation of external normal alleles into the genome of malfunctioning cells by modified viral vectors [14]. This gene addition method has been particularly successful in the treatment of monogenic disorders [15–20], allowing for recent marketing authorization of specific gene therapy products for immunodeficiencies, B-cell leukemias, lymphomas [21] and transfusion-dependent beta-thalassemia [22]. However, gene therapy’s effectiveness in the field of infectious diseases, especially those caused by intracellular infectious agents, such as *Brucella*, remains to be evaluated, since only inhibition of *Mycobacterium tuberculosis* [23, 24] and clearance of viral agents [25–28] have been reported to date.

To this end, our ultimate goal is to develop novel CRISPR/Cas9 lentiviral vectors which, after in vivo administration, would be capable of transducing the macrophages of the host, where brucellae parasitize, and inactivate specific genes that code for factors which play a critical role in their intracellular replication as an alternative therapeutic approach.

Previously, we demonstrated that ovine macrophages possess a high-level potency towards transduction under certain culturing conditions, using a green fluorescence protein (GFP) lentiviral reporter vector and a standard transduction protocol at a multiplicity of infection (MOI) of 60 [29]. We have also constructed an ovine macrophage infection model with *B.melitensis*, to mimic the host cell/microorganism interaction in vitro [30]. In this study, we proceeded to transduction of the infected cells with lentiviral vectors bearing the clustered, regularly interspaced short palindromic repeats/CRISPR-associated protein 9 (CRISPR/Cas9) technology that have been designed to inactivate genes which play a critical role in the replication of *Brucella* within the host cells, namely RNA polymerase subunit A (RpolA) or virulence-associated gene *virB10* [1]. We show that the number of internalized brucellae/cell is significantly decreased 1 and 4 days post transduction with the CRISPR/Cas9 vector against bacterial RpolA at an MOI of 60. Moreover, we show that bacterial load is not affected when macrophages are exposed to the conventional GFP lentiviral vector; a fact that underscores the bactericidal effect of the RpolA CRISPR/Cas9 system. On the contrary, the VirB10 vector demonstrated only a modest reduction in the bacterial load, suggesting that further improvements might possibly be needed towards the selection of target genes. In all cases, co-existence of brucellae and the vectors in the macrophage cytoplasm was well-tolerated and gene marking, in terms of vector copy number (VCN) was persistent throughout the culture duration. Overall, although our results may need to be further verified in vivo, may pave the way for any gene therapy application against zoonoses in the future.

## Results

### Pilot transduction of infected sheep macrophages with a conventional GFP lentiviral vector

We proceeded to a pilot transduction of the *Brucella*-infected macrophages in order to test their performance in culture, as well as their potency towards gene transfer, under conditions of heavy bacterial loads produced by the MOI of 5000. To this end, we utilized the GFP lentiviral vector that was previously shown to effectively transduce ovine macrophages at an MOI of 60 [29] and measured the VCN, as well as the number of intracellular brucellae, 1 and 4 days post transduction, that correspond to 3 and 6 days post infection with *Brucella*. We did not observe any toxic events associated with vector exposure, since, at all times, viability of macrophages, measured by trypan blue exclusion, was over 90% (Table 1), suggesting that cultured macrophages may endure concomitant infections from agents of both bacterial and viral origin. At both time points, comparison of untransduced *Brucella*-infected cells with their transduced counterparts did not yield any significant differences in bacterial loads (*Brucella* untransduced D1 vs *Brucella* + GFP D1: 447.34 ± 65.12 vs 404.94 ± 126.51, *p* = 0.57 and *Brucella* untransduced D4 vs *Brucella* + GFP D4: 276.67 ± 138.01 vs 184.85 ± 29.26, *p* = 0.24) (Table 2), indicating that exposure to a vector expressing a fluorescent protein does not suffice to clear engulfed brucellae from the host cytoplasm. These observations were further consolidated by analyzing the colony-forming units (CFU) produced from 500 infected macrophages that were lysed and seeded in sheep blood agar plates (Table 2).

**Table 1:** The absolute number of live macrophages per well during the experiment, measured by trypan blue exclusion

		D1 post transduction	D4 post transduction
*Brucella* untransduced	Exp 1	75,110	110,000
	Exp 2	72,125	101,975
	Exp 3	70,000	102,500
	Exp 4	84,323	100,075
	Average	75,389.5	103,637.5
	SD	6313.7	4367.7
*Brucella* + GFP	Exp 1	74,000	105,000
	Exp 2	73,500	101,700
	Exp 3	68,000	98,000
	Exp 4	78,575	105,000
	Average	73,518.7	102,425
	SD	4330.2	3335
*Brucella* + VirB10	Exp 1	85,000	99,700
	Exp 2	73,000	110,000
	Exp 3	81,500	103,400
	Exp 4	76,500	108,575
	Average	79,000	105,418.7
	SD	5307.2	4751.4
*Brucella* + RpolA	Exp 1	76,580	106,000
	Exp 2	80,475	107,800
	Exp 3	90,000	110,350
	Exp 4	76,000	99,900
	Average	80,763.7	106,012.5
	SD	6470.1	4448.6

All experimental conditions were initiated by seeding 50,000 macrophages per well. Untransduced, *Brucella*-infected cells served as a control

**Table 2:** Detailed results from all the experiments performed to determine the number of incorporated brucellae after transduction with the various lentiviral vectors (CFU assay from 500 infected and lysed macrophages seeded in blood agar dishes and from 50,000 infected macrophages analyzed by Real-Time PCR)

		D1 post transduction		D4 post transduction	
		Total number of colonies (agar)	Brucellae/cell (Real-Time PCR)	Total number of colonies (agar)	Brucellae/cell (Real-Time PCR)
*Brucella* untransduced	Exp 1	55	412.69	35	210.09
	Exp 2	64	488.82	36	483.57
	Exp 3	51	513.84	40	211.50
	Exp 4	60	374.00	43	201.51
	Average	57.5	447.34	38.5	276.67
	SD	5.68	65.12	3.69	138.01
*Brucella* + GFP	Exp 1	66	243.54	32	141.28
	Exp 2	57	371.39	33	195.71
	Exp 3	53	472.10	40	198.36
	Exp 4	50	532.71	39	204.05
	Average	56.5	404.94	36	184.85
	SD	6.95	126.51	4.08	29.26
*Brucella* + VirB10	Exp 1	30	259.82	21	181.39
	Exp 2	32	Undetermined	28	133.04
	Exp 3	31	318.92	30	156.65
	Exp 4	37	228.00	23	125.00
	Average	32.5	268.91	25.5	149.02
	SD	3.1	46.14	4.2	25.42
*Brucella* + RpolA	Exp 1	22	Undetermined	15	161.01
	Exp 2	26	221.09	18	139.49
	Exp 3	22	161.78	20	65.47
	Exp 4	23	107.84	11	60.42
	Average	23.25	163.57	16	106.6
	SD	1.89	56.65	3.91	51.21

The standard curves produced by quantitative Real-Time PCR were used to extrapolate the absolute number of brucellae and endogenous ovPrp copies per reaction. Brucellae/cell were ultimately calculated by normalizing the absolute number of brucellae to the ovPrp copies. Untransduced, *Brucella*-infected cells served as a control

To complete the analysis, we calculated the viral copy number per cell also by Real-Time PCR. Our results showed that, in average, there was an anticipated ~ 50% drop in the VCN of the GFP-transduced, *Brucella*-infected macrophages between the two time points post transduction (Table 3), which, stands in accordance with our previous findings [29].

**Table 3:** Detailed results from all the experiments performed to determine the vector copy number (VCN)/cell after transduction with the various lentiviral vectors

		VCN/cell D1 post transduction	VCN/cell D4 post transduction
*Brucella* untransduced	Exp 1	0.05	0.14
	Exp 2	0.02	0.05
	Exp 3	0.12	0.04
	Exp 4	0.00	0.01
	Average	0.05	0.06
	SD	0.05	0.06
*Brucella* + GFP	Exp 1	5.15	6.21
	Exp 2	6.76	2.48
	Exp 3	22.47	14.02
	Exp 4	23.93	7.80
	Average	14.58	7.63
	SD	10.00	4.81
*Brucella* + VirB10	Exp 1	0.80	0.30
	Exp 2	15.24	11.94
	Exp 3	1.84	0.13
	Exp 4	14.24	10.39
	Average	8.03	5.69
	SD	7.77	6.35
*Brucella* + RpolA	Exp 1	11.07	3.94
	Exp 2	4.19	11.89
	Exp 3	9.91	4.89
	Exp 4	22.85	8.52
	Average	12.01	7.31
	SD	7.83	3.64

The standard curves produced by quantitative Real-Time PCR were used to extrapolate the absolute number of vector and endogenous ovPrp copies per reaction. VCN/cell was ultimately calculated by normalizing the absolute number of vector copies to the ovPrp copies. Untransduced, *Brucella*-infected cells served as a control

### The CRISPR/Cas9 vector against bacterial RpolA results in a significant decrease of intracellular brucellae within host macrophages of ovine origin

**Fig. 1:**
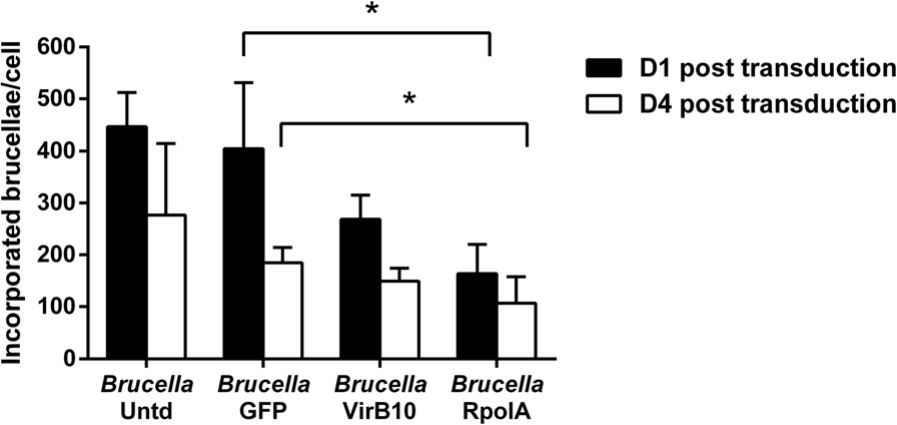
Comparative analysis of incorporated brucellae per cell in ovine macrophages infected with *Brucella* at MOI = 5000, 1 and 4 days post transduction with different vectors at MOI = 60 (quantitative Real-Time PCR). Each experimental condition was repeated 4 times. Untd: untransduced; GFP: green fluorescence protein; VirB10: virulence-associated gene *virB10*; RpolA: RNA polymerase subunit A; *p < 0.05 (one-way ANOVA). All results are expressed as means ± standard deviation (SD)

To adequately assess any decrease in the bacterial load resulting from the potential therapeutic effect of our CRISPR/Cas9 vectors, we chose to proceed in transducing macrophages infected with the highest possible macrophage: *Brucella* ratio (MOI = 5000), based on our previous work [30]. We utilized two different vectors; one targeting and inactivating *Brucella*’s virulence-associated gene *virB10* and another against the *rpolA* gene. At both time points post transduction, brucellae were significantly reduced in the macrophage cohort treated with the RpolA vector, as compared with their GFP-transduced counterparts (*Brucella* + GFP D1 vs *Brucella* + RpolA D1: 404.94 ± 126.51 vs 163.57 ± 56.65, *p* = 0.03 and *Brucella* + GFP D4 vs *Brucella* + RpolA D4: 184.85 ± 29.26 vs 106.6 ± 51.21, *p* = 0.04) (Fig. 1, Table 2). Interestingly, this was not the case with the VirB10 vector, although the numbers of intracellular brucellae appeared reduced (*Brucella* + GFP D1 vs *Brucella* + VirB10 D1: 404.94 ± 126.51 vs 268.91 ± 46.14, *p* = 0.14 and *Brucella* + GFP D4 vs *Brucella* + VirB10 D4: 184.85 ± 29.26 vs 149.02 ± 25.42, *p* = 0.11) (Fig. 1, Table 2). These data indicate that surplus amelioration in the VirB10 vector design might be needed and in addition, cells may need to be co-cultured with the vectors with higher MOI or for longer periods of time in order to further decrease bacterial loads in both vector cohorts.

Gene marking, in terms of vector copy number, demonstrated that viral copies were reduced, though not significantly, between Days 1 and 4 in culture, as observed with GFP-transduced macrophages (Table 3). The VirB10 group presented a slightly lower VCN/cell at both time points, compared with its RpolA counterparts that presented an almost identical VCN with the GFP-transduced cells. This might explain, in part, VirB10 vector’s modest bactericidal properties, that may be overcome in the future by exposing the cells to even higher MOIs.

## Discussion

The “test and slaughter” programs, for the control and eradication of brucellosis [7] have been failing in southern Europe for more than 40 years and alternatives are necessary to be found [12]. Widespread prevalence of *Brucella* species among ruminants poses safety concerns not only towards traditional dairy products, but also outlines a substantial financial burden for both the European Union and animal breeders, due to reimbursement costs [13]. Additionally, the live vaccine REV-1 that is utilized for animal immunization, is not entirely safe for practitioners and may also be causative of undesired side effects in livestock [9].

For the first time, we have implemented a state-of-the-art method, which has been previously clinically effective in other approaches [15–20], in order to treat a bacterial infection affecting livestock, with a direct benefit in humans. Gene targeting, namely the in situ alteration of genes by specific nucleases, such as those of the CRISPR/Cas9 system [31], represents a novel strategy which, owing to the nuclease-associated creation of double stranded breaks in the DNA, replacement, insertion, or deletion of a sequence in a certain locus may be achievable. This procedure is rendered feasible either by homologous recombination, or by non-homologous end-joining and represents an incremental approach to the simple gene addition protocols applied until recently. The CRISPR/Cas9 technology, has been successfully tested at a clinical level against hepatitis B virus (HBV), aiding in its clearance [25], and against human immunodeficiency virus-1 (HIV-1), by inactivating the expression and replication of its genes [26]. Ever since, many more successful preclinical applications have been reported for *Mycobacterium tuberculosis*, hepatitis C virus (HCV), as well as herpes simplex virus (HSV) [32]. Consequently, the CRISPR/Cas9 system could potentially overcome the hurdles posed for the eradication of brucellosis, through the development of a molecular therapy that may clear brucellae within the cells where they parasitize, such as macrophages, by introduction, via a systemically administered lentiviral vector, of a nuclease genetic information that would provoke lesions in specific genetic features of the microorganism, without harming the host DNA.

Previously, we created a cellular model, using macrophages from sheep peripheral blood to simulate infection with *Brucella* [30]. Subsequently, and since we have previously reported that ovine macrophages may be effectively transduced by a GFP reporter vector at the relatively high MOI of 60 [29], we sought to determine whether the cells could survive concomitant infections of bacterial and viral origin and, if transduction itself, even with a reporter vector, may lower bacterial loads. At both Day 1 and Day 4 post transduction, *Brucella* numbers in GFP-transduced macrophages were not drastically lowered when compared to their untransduced counterparts. However, when brucellae measured in each cohort on Day 1 and Day 4 were compared together, they presented a decrease in both untransduced and GFP-transduced cohorts (*Brucella* untransduced D1 vs *Brucella* untransduced D4: 447.34 ± 65.12 vs 276.67 ± 138.01, *p* = 0.06 and *Brucella* + GFP D1 vs *Brucella* + GFP D4: 404.94 ± 126.51 vs 184.85 ± 29.26, *p* = 0.01). This, as already mentioned above, is an anticipated phenomenon, since it has been previously shown that the *Brucella* load in cultured macrophages is been naturally reduced over time [33]. We also implemented the CFU assay to determine intracellular bacterial loads. However, due to the slow growth of the bacterium, isolation and culture of *Brucella* by routine agar methods can be particularly challenging. Based on our previous results [30], probably because not all incorporated brucellae can grow in culture after lysis of host cells or because intracellular brucellae are not viable anymore, our CFU assay generates lower numbers of intracellular brucellae/cell than Real-time PCR.

Exposure of infected macrophages to the CRISPR/Cas9 vector against *Brucella*’s RpolA, revealed an instant bactericidal effect, since the bacterial load was significantly reduced, starting from 24 h, and persisted until Day 4 post transduction. Compared to the GFP-transduced cells, the RpolA system conferred an additional decrease to brucellae by 50% on Day 1 and by 9% on Day 4 post transduction with an average of 12.01 and 7.31 vector copies per cell respectively. Consequently, the bactericidal effect was presented much milder on Day 4 than the one observed on Day 1. This may be attributed to silencing or clearance of the vector over time and it could be overcome by exposing the macrophages to higher MOIs than 60 that was implemented here. This could counterbalance the failure of ovine macrophages to maintain a sustained VCN/cell after a certain period of time [29], should we decide to culture them for longer periods of time in order to attempt a further decrease in bacterial loads. On the contrary, the VirB10 vector showed a modest decline in the number of incorporated brucellae at both time points suggesting that the nature of the target gene has some importance in the final therapeutic outcome. Given that both vector batches were generated the same day and under the same experimental conditions, we conclude that the somewhat decreased performance of the VirB10 vector is due to the different guide RNA compared to the RpolA vector and not because of inconsistencies during the vector manufacturing process. Indeed, it has been previously shown that mutant brucellae which lacked the *virB10* gene and were therefore, deficient in their Type 4 secretory pathway, still invaded host cells and interacted with the early and late endosomes in a manner similar to that by wildtype bacteria. However, after a certain amount of time, they were ultimately engulfed by lysosomes and targeted for degradation [34]. Therefore, it may be possible that lack of the RNA polymerase enzyme confers more direct toxic effects to brucellae than disruption of the Type 4 secretory pathway, which may require a length of time that extends beyond the design of our assay in order to be evident.

Moreover, in this study, owing to the low vector titers (1 × 10^7^ infectious units/ml) we were unable to proceed with transductions at MOIs higher than 60. Therefore, it is possible that improvements in vector production procedures, alterations in the vector constructs in terms of different genes as targets or even pseudotyping with alternative envelope glycoproteins may aid in the manufacturing of high-titer viral supernatants in the future that would allow us to proceed to transduction at more elevated MOIs and assess whether brucellae may be completely cleared from the host macrophages.

## Conclusion

In summary, this is the first report of such a therapeutic approach and the results produced here may be further consolidated after administration of the vectors in animal models of brucellosis in the future. However, our data in total can be helpful not only towards promoting a holistic sanitation of livestock, but also towards advancing the modernization of the agricultural economy, as well as the protection of the breeders’ income. Finally, this study may prove critical for the treatment of human brucellosis and provide the basis for the implementation of gene therapy for other infectious diseases as well.

## Methods

### Ovine macrophages and bacteria

Ovine macrophages were obtained by culturing the mononuclear cell fraction of sheep peripheral blood after ficoll density gradient centrifugation [29]. *Brucella melitensis* 16 M strain (ATCC 23456) was purchased from Culture Collections Public Health England (Salisbury, United Kingdom)*.* Brucellae were cultured as previously described [30]. Briefly, *Brucella* master seed was obtained by aerobically culturing purchased bacteria both on *Brucella* agar (Oxoid, Hampshire, United Kingdom) and Columbia agar sheep blood plates (Oxoid, Hampshire, United Kingdom) for 3 days at 37^ο^ C. Vials containing 1 × 10^8^ CFU/ml *B.melitensis* in brain heart broth (Oxoid, Hampshire, United Kingdom) with 15% glycerol (Sigma-Aldrich, St. Louis, MO, USA) were screened for contamination and stored at -80^o^ C. *Brucella* working seed was prepared by spreading bacteria from the master seed vial on Columbia agar sheep blood plates. Vials of working seed were prepared following the same protocol for the master seed.

To infect macrophages at particular MOI, 10-fold serial dilutions of freshly cultured bacteria were plated on Columbia agar sheep blood plates and CFUs were determined after 3 days to estimate bacterial concentrations per ml. MOI was controlled by titration of the bacteria utilizing a suspension turbidity detector (Liophichem, Teramo, Italy).

### Lentiviral vector production and titration

The construct of the reporter, GFP-encoding lentiviral vector containing the human phosphoglycerate kinase (PGK) promoter and the 1.2-kb cHS4 insulator inserted in the deleted region of U3, has been previously published [35]. The novel CRISPR/Cas9 lentiviral vectors were constructed with standard cloning procedures [36], by utilizing guide RNAs for the targeted suppression of the *B.melitensis virB10* and *rpolA* genes. The plasmid utilized in both vectors (lentiCRISPR v2, Addgene, Cambridge, MA, USA), contained two expression cassettes; hSpCas9 and the chimeric guide RNA. The vectors were digested with BsmBI, and a pair of annealed oligos was cloned into the sgRNA scaffold. The oligos were designed based on ~ 20-bp long target site sequences, from the *B.melitensis virB10* and *rpolA* genes, and were flanked on the 3′ end by a 3-bp NGG protospacer adjacent motif (PAM) sequence. The expression of the target sequences was driven by a universal U6 promoter. The sequence for *virB10* gene is from *Brucella melitensis* 16 M chromosome II, complete sequence, GenBank: AE008918.1, position: 33406–34,203 and the sequence for *rpolA* is from *Brucella melitensis* 16 M chromosome I, complete sequence, GenBank: Genbank Accession No: CP007763.1 from position 656,835–657,848.

All lentiviral vectors used in this study were self-inactivating (SIN), pseudotyped with vesicular stomatitis virus glycoprotein (VSV-G) and produced by transfection of 293 T cells with calcium phosphate precipitation using standard procedures [37]. For titration, the 100-fold vector concentrates produced by ultrafiltration were serially diluted and used to infect mouse erythroleukemia cells (MEL-585) as previously described [37]. Determination of the viral titer was calculated with flow cytometry per viral dilution, either by analyzing the percentage of GFP positive cells or the percentage of Cas9 positive cells after intracellular staining for the Cas9 nuclease as previously described [35], using the antiCRISPR-Cas9 antibody (Abcam, Cambridge, UK) as the primary antibody and then the mouse IgG-FITC (BD, Franklin Lakes, NJ, USA) as the secondary antibody.

### Rationale for the generation of Brucella guide RNAs

In order to disrupt intracellular survival of *Brucella*, we searched through its genome to identify most necessary genes. Between various genes that were identified, through attenuation mutagenesis studies, to play important roles in survival of this pathogen, we chose Type 4 secretory pathway proteins, which are encoded by the *virB* operon, and the alpha subunit of the pathogen’s housekeeping RNA polymerase [38, 39] as our final candidates. Type 4 secretion system (T4SS) is composed of 12 proteins (VirB1-VirB12), all encoded by the *virB* operon. T4SS proteins assemble a channel through which virulence factors translocate into the host cell. These factors play crucial roles in the pathogen’s intracellular survival, manipulation of the host cell immune response and vehicle trafficking [34]. As the VirB10 protein shows to participate in the assembly of critical channel domains, we decided to target its coding sequence for our CRISPR/Cas9 application. RpolA was chosen because of its necessity during transcription. That means, disruption of this protein most likely would attenuate pathogen proliferation.

In order to select guide RNAs, we searched through the coding regions of the *virB10* and *rpolA* genes to find sequences of 17–24 nucleotides, ending next to a PAM sequence (5′-NGG-3′), having their GC content between 40 and 80%, and being located close to the 5′ end, as the production of insertions/deletions at this region would provoke an early shift of the open reading frame during translation and would efficiently disrupt the proteins’ function. Moreover, we performed BLAST searches, in order to ensure that our guide RNAs would not interact with the host cell genome. As per the aforementioned, the guide RNAs selected were the 5′-GUCGUCACCAAGUCCAGCGGCGAUACGG-3′ for the *virB10* gene and the 5′-GUGACCGCUGUCCAGAUCGACGG-3′ for the *rpolA* gene.

### Lentiviral transduction of macrophages infected with Brucella

Ovine macrophages were seeded in 24-well tissue culture plates at 5 × 10^4^ cells/ml. Twenty-four hours later, the cells were exposed to Brucella at an MOI of 5000 in an antibiotic-free macrophage-specific medium [29]. The next day, cells were washed and cultivated in the presence of 5% penicillin/streptomycin (Invitrogen, Carlsbad, CA, USA) and 0.5 mg/ml lysozyme (Roche, Basel, Switzerland). After killing extracellular bacteria overnight, cells were transduced with concentrated GFP or CRISPR/Cas9 vector stocks corresponding to an MOI of 60. Twenty-two μg/ml hexadimethrine bromide (Polybrene, Sigma-Adrich, St. Louis, MO, USA) were added to the cells to enhance gene transfer at the time of vector exposure. Ultimately, after transduction, cells were further kept in culture for 1 and 4 more days. At all time points of the procedure, viability of macrophages was maintained by changing the cell culture medium and assessed by trypan blue exclusion. In order to maintain the culture supernatant at a constant level, all wells were supplemented with the required fresh medium volume.

At the days of harvest, cells were detached from the culture plates and lysed as previously described [30]. Transduction efficiency was assessed by quantitative Real-time PCR with vector-specific primers for the determination of the VCN per cell. Untransduced cells infected with Brucella served as control at all times.

**Fig. 2:**
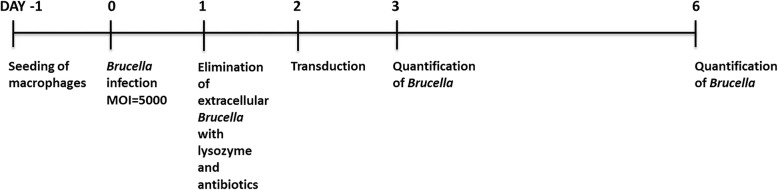
Schematic diagram and timeline of the study

At all time points, the presence or absence of live brucellae in the culture supernatant, as well as the supernatant collected from the final cell wash, was verified by seeding the old culture media in Columbia agar sheep blood plates [30]. To assess intracellular live brucellae, 500 infected cells were lysed by adding 0.1% saponin (Sigma-Aldrich, St. Louis, MO, USA) and plated in Columbia agar sheep blood plates as previously described [30]. The experimental design is graphically outlined in Fig. 2.

### Real-time PCR analysis of Brucella and vector copies in macrophages

Extraction of genomic DNA from cultured cells was conducted with the High Pure PCR template preparation kit (Roche, Basel, Switzerland). Internalized brucellae were quantified with the *Brucella* genus Genesig advanced kit (Primerdesign, Chandler’s Ford, United Kingdom). Lentiviral VCN was analyzed with vector-specific primers for the Rev. response element (RRE) as previously described [37, 40]. The endogenous ovine prion protein (ovPrp) single-copy chromosomal gene [41, 42] was utilized to adjust for equal loading of *Brucella*- and vector-containing DNA as previously described [29]. Construction of the standard curves for the quantification of brucellae and vector copies was already described elsewhere [30].

### Statistics

Multiple comparisons were performed using the one-way ANOVA. Values of *p* < 0.05 were considered statistically significant. All results are expressed as means ± standard deviation (SD).

## Data Availability

The datasets used and/or analyzed during the current study are available from the corresponding author on reasonable request.

## Abbreviations

CRISPR/Cas9: Clustered, regularly interspaced short palindromic repeats/CRISPR-associated protein 9
GFP: Green fluorescence protein
HBV: Hepatitis B virus
HCV: Hepatitis C virus
HIV-1: Human immunodeficiency virus-1
HSV: Herpes simplex virus
MEL-585: Mouse erythroleukemia cells
MOI: Multiplicity of infection
ovPrp: Ovine prion protein
PAM: Protospacer adjacent motif
PGK: Human phosphoglycerate kinase
RpolA: RNA polymerase subunit A
RRE: Rev. response element
SD: Standard deviation
SIN: Self-inactivating
T4SS: Type 4 secretion system
Untd: Untransduced
VCN: Vector copy number
VirB10: Virulence-associated gene *virB10*
VSV-G: Vesicular stomatitis virus glycoprotein
